# Barriers to interventional pain management in patients with cancer: a systematic review

**DOI:** 10.1186/s12904-026-02235-5

**Published:** 2026-07-14

**Authors:** Elham S. Alzahrani, Asrar O. Abduldaiem, Abdulrahman I. Alardan, Njood A. Alshowigi

**Affiliations:** Department of Oncology, Prince Sultan Military Medical City, Riyadh, Saudi Arabia

**Keywords:** Interventional Pain Management, Barriers, Palliative Medicine, Systematic Review, Meta-Analysis

## Abstract

**Background:**

Systemic opioids fail to provide adequate relief in 10–30% of advanced cancer patients due to poor response or debilitating adverse effects. While intrathecal drug delivery and neurolytic blocks offer effective alternatives, these interventions are severely underutilized due to systemic barriers, inadequate provider education, and late referrals, leaving pain undertreated in up to 40% of patients. This review aims to compile barriers to pain management interventions among patients with terminal cancer.

**Methods:**

We searched for relevant articles up to February 2026 from PubMed, Scopus, and Web of Science. Two independent reviewers extracted data from the selected studies, including baseline information, outcomes, interventional pain management methods, patient data, physician and facility data, and barriers to different pain management interventions.

**Results:**

We collected 7,614 records after excluding 3,529 duplicates. Thorough screening resulted in the retrieval of 14 entries eligible for inclusion in our review. Our synthesis from 14 studies identified multi-level barriers to interventional pain management, including Intrathecal Drug Delivery Systems (IDDS) and Celiac Plexus Neurolysis (CPN). Patient-related barriers centered on lack of understanding, as well as fear of adverse effects and infection. Clinician-related obstacles were significant; 51% of pain fellows reported unmet training needs, while only 28% of hospice providers felt confident managing IDDS, leading over 80% to prefer systemic analgesics. Systemic issues included fragmented care coordination, a median two-year delay from diagnosis to IDDS implant, and a lack of standardized guidelines. Furthermore, socioeconomic disparities were evident, as racial minorities and residents of lower-income neighborhoods faced significantly lower access to these interventions.

**Conclusion:**

The underutilization of interventional pain management techniques is driven by a complex interplay of patient misconceptions, clinician training gaps, and systemic fragmentation.

**Supplementary Information:**

The online version contains supplementary material available at https://doi.org/10.1186/s12904-026-02235-5.

## Introduction

Despite the WHO analgesic ladder achieving effective pain control in 70% to 90% of oncology patients, moderate to severe pain persists in approximately 80% of individuals with advanced-stage cancer [1, 2]. Standard pharmacological regimens have an inherent failure rate of 10% to 30%, leaving a subset of patients with refractory pain unresponsive to both non-opioid and opioid analgesics [2]. Multiple clinical evaluations estimate that systemic therapies fail to provide acceptable relief in 10% to 20% of patients with advanced disease, with data on opioid response variability revealing that up to 26% of those treated with strong opioids are poor or non-responders, achieving less than a 30% reduction in pain intensity after four weeks of treatment [3–7]. For those who do respond, chronic high-dose opioid therapy is limited by debilitating, dose-limiting adverse effects that compromise both adherence and quality of life [8]. These complications range from common somatic issues, such as intractable constipation, nausea, vomiting, pruritus, and urinary retention, to severe neurological and cognitive impairments that hinder dose escalation [9–13]. At elevated doses, systemic opioids can also precipitate life-threatening respiratory depression, withdrawal syndromes, and opioid-induced neurotoxicity characterized by myoclonus, hyperalgesia, and seizures, the latter being exacerbated in patients with renal impairment due to the accumulation of neurotoxic metabolites [10, 13].

Among interventional strategies for refractory cancer pain, intrathecal drug delivery systems (IDDS), celiac plexus neurolysis (CPN), and vertebral augmentation currently hold the strongest level of clinical evidence (Grade I-A), while splanchnic nerve neurolysis (Grade I-B) and surgical cordotomy (Grade II-B) offer additional options for intractable abdominal and unilateral nociceptive pain, respectively [1]. These interventions enhance quality of life by providing targeted analgesia that circumvents the need for high-dose systemic opioids, thereby mitigating debilitating adverse effects such as sedation, cognitive impairment, nausea, and severe constipation [8, 9]. By depositing medications directly into the cerebrospinal fluid, intrathecal systems bypass the blood-brain barrier and first-pass hepatic metabolism, achieving maximal pharmacodynamic efficiency at dorsal horn receptors with minimal peripheral exposure [8]. This approach restores functional capacity, mobility, and emotional well-being while enabling patients to continue life-prolonging oncological treatments [8, 9]. Likewise, neurolytic blocks interrupt nociceptive and sympathetic pathways, conferring long-lasting analgesic effects that prevent quality-of-life deterioration and reduce opioid-induced adverse events [1, 7].

Despite this evidence base, a disconnect remains between clinical recommendations and real-world utilization [2, 3]. Observational data indicate that cancer pain remains undertreated in up to 40% of patients, yet interventions such as IDDS are implemented in only a small minority of eligible individuals across various healthcare systems [2, 3, 11]. An analysis of the United States Nationwide Inpatient Sample unveiled drastically low procedural rates for IDDS implantation among oncology patients, driven by systemic disparities and inadequate provider education [3]. Japanese national surveys also demonstrated that interventional procedures were available at only 30.7% to 54.8% of designated cancer hospitals, with median procedural volumes restricted to merely one to four cases over a three-year period [14]. Multiple studies have documented the underutilization, systemic barriers, and delayed referral patterns that characterize interventional pain management in palliative and oncological settings [15–17]. Primary oncologists frequently underutilize pain specialists, with patients referred to specialized refractory pain clinics only in the terminal phases of disease when functional status has already deteriorated [5]. Persistent fragmentation of care pathways further compounds these challenges, as pain specialists are consulted too late, whereas oncologists and home hospice physicians remain unfamiliar with available interventional techniques [16, 18].

Mapping implementation barriers across patient, provider, and systemic levels is essential because these barriers are tied to the socio-cultural context in which pain perception, manifestation, and treatment occur [19]. For example, pinpointing the root causes of procedural cancellations allows healthcare facilities to prioritize their improvement efforts and decrease cancellation rates through targeted operational strategies [20]. Moreover, addressing these obstacles enables the creation of innovative clinical models that use intensive patient education, shared decision-making, and professional peer feedback to overcome treatment barriers [21, 22]. Ultimately, resolving these barriers through pragmatic, evidence-informed actions optimizes timely, integrated care and substantially improves patient outcomes [16]. Currently, available literature lacks a comprehensive synthesis of such barriers. This systematic review aims to accrue and map all reported barriers to the use of interventional pain modalities in cancer patients.

## Methods

In accordance with the guidelines provided by the Cochrane Handbook for Systematic Reviews of Interventions and the Preferred Reporting Items for Systematic Reviews and Meta-Analyses (PRISMA), we conducted a systematic synthesis of the documented barriers to the implementation of pain management procedures for cancer patients [23, 24].

### Eligibility criteria

Inclusion Criteria:

- Cross-sectional articles exploring barriers to interventional pain management in patients with cancer.
- Studies assessing active clinical environments, practicing healthcare providers, or patients.
- Relevant articles reported in English.
- Articles which could be downloaded in full text.

### Exclusion criteria

- All letters to editors, opinions, and comments.
- Abstracts and reviews.

### Information sources and search strategy

A multi-step process was used to retrieve records and gather evidence on barriers to pain management modalities among cancer patients. Initially, a broad search was performed in PubMed, and Scopus using general terms to identify preliminary articles. All collected terms were then individually assessed for relevance, and any redundant terms were removed. This refined search strategy, which included MeSH terms and relevant keywords, was subsequently applied systematically to PubMed, Scopus, and Web of Science. The final search strategy was as follows: (Pallia* OR Hospice* OR Terminal* OR Neoplasm* OR Cancer* OR “End of life” OR Metastatic* OR Oncolog) AND (“Nerve Block” OR (anesthesia AND epidural) OR (injection AND spinal) OR Intrathecal OR “Interventional pain” OR Neurolysis OR Cordotomy OR “Radiofrequency ablation” OR Sympathectomy OR Vertebroplasty OR Kyphoplasty OR Cementoplasty OR “Celiac plexu” OR Splanchnic OR Neuromodulation OR “Ganglion block”) AND (Consultation OR Referral* OR Barrier* OR Access* OR Delay* OR Refusal* OR Underutiliz* OR Availabl* OR “Wait times” OR Gatekeeping OR Reimbursement OR Insurance OR Education* OR Attitude). The search was completed in February 2026 using this specific, detailed query. Additionally, forward and backward searches were conducted using the records obtained from the initial strategy to identify further relevant studies.

### Selection process

To minimize bias, the senior author anonymized the records obtained through the search strategy by removing author names and any identifying information. Two reviewers then independently conducted blind screening of the titles and abstracts of the anonymized records based on a predefined checklist. In cases of conflict, the senior author intervened to resolve discrepancies. After the initial screening, the same two blinded reviewers evaluated the full texts of the selected articles for eligibility, comparing their assessments to address any remaining disagreements.

### Data collection

In accordance with predefined criteria, full-text articles were examined for relevant data, which were subsequently extracted into a shared Google spreadsheet. The initial phase involved collecting baseline information, such as the author’s last name, country of origin, and study outcomes. Additional details were gathered, including patient sample size, age, gender distribution, cancer type, functional status, metastatic status, procedure type, perceived barriers, specialties, experience levels, and facility type. Narrative synthesis centered on barriers to pain management interventions for cancer patients, categorized as patient-related, clinician-related, or systemic barriers.

### Outcome measures

#### Barriers to pain management

We synthesized data on the barriers that prevent the implementation of various pain management procedures, such as intrathecal drug delivery systems (IDDS) or celiac plexus neurolysis (CPN). These included patient-related barriers, which center on the psychological, informational, and physical concerns of the patients themselves, individual patient circumstances, and other patient-related drivers for procedure cancellations or delays. In addition, we documented clinician-related factors, such as expertise, attitudes, and educational issues. Systemic issues, including structural frameworks of the healthcare system as well as logistical barriers, were also addressed.

### Risk of bias

A quality assessment of the records included was performed using the NIH Quality Assessment Tool for Observational and Cohort Studies [25]. Before the formal assessment, a calibration process was carried out in which two independent reviewers piloted the tools on five randomly selected studies, discussed discrepancies, and refined the application criteria to ensure consistent interpretation. All subsequent evaluations were conducted independently by two reviewers, with any disagreements resolved by a third reviewer. During the appraisal, an important observation was made regarding the NIH tool. Although it offers a robust framework for assessing bias, four of its fourteen domains are inherently not applicable to cross-sectional studies. As a result, studies rated as “Fair” under this tool would likely have received a “Good” rating if these design-specific domains had been excluded from the scoring calculation.

## Results

### Study selection

**Fig. 1:**
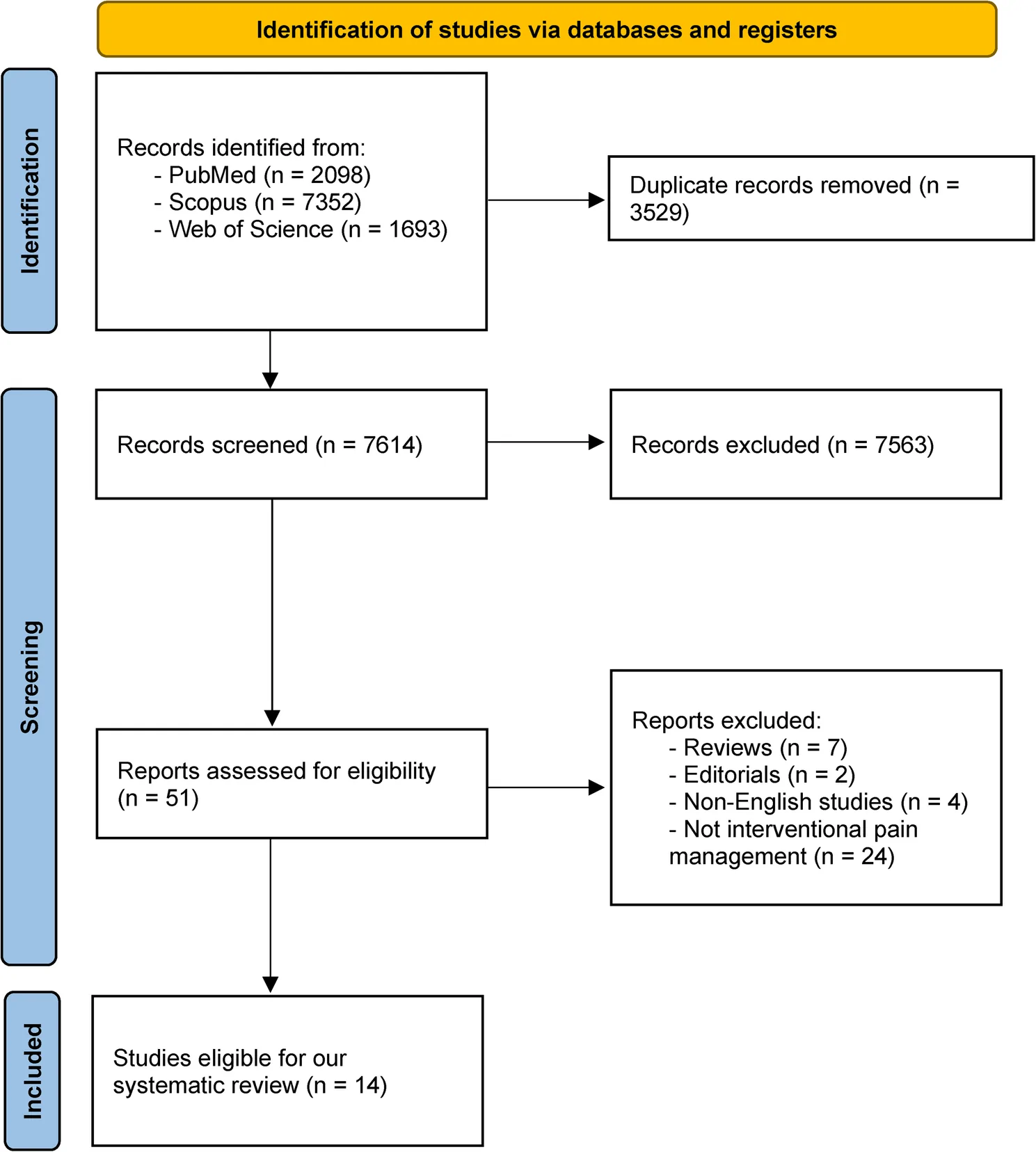
PRISMA Flow Diagram of the Study Selection Process

A search of three databases yielded a total of 11,143 records. After removing 3,529 duplicates, 7,614 unique records remained. These records underwent title and abstract screening, resulting in the exclusion of 7,563 entries. The full texts of the remaining 51 records were retrieved and assessed against the eligibility criteria. During this evaluation, 37 records were excluded: four non-English articles, seven review articles, two editorial comments, and 24 studies that did not provide data on pain management interventions. Eventually, 14 articles were included in the review. The study selection process is illustrated in the flow diagram presented in Fig. 1.

### Characteristics of the included studies

This systematic review included 14 cross-sectional studies [2, 3, 5, 11, 13–15, 17, 18, 20, 26–29], with a combined sample size of 27,837 participants. Age was reported in three studies, with mean ages ranging from 58.8 to 61 (12–13.7) years. Five studies reported gender distribution, and 12,308 patients were male. Cancer types were diverse, with lung cancer being the most frequently reported (ranging from 8% to 27.15% across studies), followed by gastrointestinal cancers (including colorectal, pancreatic, and liver cancers), breast cancer, genitourinary cancers, and sarcomas. One study exclusively focused on pancreatic cancer [26]. Functional status was assessed using various scales including ECOG performance status, ASA physical status classification, and ambulatory status, with most patients demonstrating moderate to poor functional capacity (ECOG 2–3 or ASA III–IV). Regarding interventional procedures, studies evaluated pain management procedures like intrathecal drug delivery systems (IDDS), epidural opioid analgesia, neurosurgical ablative procedures (cordotomy, cingulotomy, mesencephalotomy), celiac plexus neurolysis (CPN), and others. Provider and facility survey variables revealed that participating healthcare professionals represented multiple specialties including pain medicine, anesthesia, palliative care, oncology, neurosurgery, internal medicine, surgery, and hospice care, with experience levels ranging from fellows to senior consultants and medical directors. Facility types encompassed university hospitals, tertiary care centers, district/general hospitals, mixed practice settings, academic training programs, and home hospice services. A complete depiction of baseline characteristics is displayed in Table 1.

**Table 1:** Summary and baseline characteristics of the included studies

Study ID			Patient & Clinical Data Variables								
	Sample Size	Country	Age, mean (SD)	Gender, Male, *N* (%)	Cancer Type	Functional Status	Metastatic Status	Active Treatment	Procedure Type	Perceived Barriers	Proportion of patients who refused palliative
Barnosky 2025 [2]	18	UK	NA	NA	NA	NA	NA	NA	Intrathecal Drug Delivery System	NA	NA
Cahana 2007 [15, 15]	32	Switzerland	NA	NA	NA	NA	NA	NA	Intrathecal and Epidural Opioid Analgesia	NA	NA
Copley 2025 [27]	164	England, Scotland, and Wales	NA	NA	NA	NA	NA	NA	NS	NA	NA
Goel 2023 [3]	22,895	USA	NR	11,345 (49.6%)	1- Lung: 6,215 (27.15%) 2- Colorectal: 5,695 (24.87%) 3- Liver: 3,755 (16.4%) 4- Bone: 1,835 (8.01%)	1- Hypertension: 12,035 (52.57%) 2- Hyperlipidemia: 7,755 (33.87%) 3- Diabetes: 4,475 (19.55%) 4- Pulmonary Disease: 3,325 (14.52%)	NR	NR	Intrathecal Drug Delivery System	1- Demographic & Economic Imbalance: The cohort was predominantly White (75.82%; *n* = 17,360), with minimal Black representation (6.46%; *n* = 1,480). Over 31% (*n* = 7,040) occupied the highest income quartile, indicating a bias toward high-socioeconomic status 2- inadequate professional education and a lack of specialized training. 3- misconceptions regarding opioid therapy	NR
Khashan 2024 [5]	207	Israel	61 (12.3)	108 (52%)	1- Lung: 49 (24%) 2- Gastrointestinal: 32 (15%) 3- Genitourinary: 29 (14%) 4- Breast: 27 (13%) 5- Sarcoma: 23 (11%)	1- ECOG 0–1 (Active): 26 (12%) 2- ECOG 2 (Up > 50%): 95 (46%) 3- ECOG 3 (Confined > 50%): 67 (32.3%) 4- ECOG 4 (Disabled): 19 (9.1%) 5- Ambulation: 64 (31%)	192 (92–93%)	137 (66%)	Neurosurgical Ablative Procedures (Cordotomy, Cingulotomy, Mesencephalotomy)	1- Lost to follow-up (*n* = 31; 15%) 2- Patient refusal (*n* = 13; 6%) 3- Died prior to procedure (*n* = 6; 3%) 4- Insufficient physician knowledge 5-Fear of the procedure 6-Logistical and travel difficulties	100 (48.3%)
Kim 2020 [20]	350	South Korea	59.6 (13.7)	89 (41.8%)	1- Upper GI (Pancreas, Liver, etc.): 77 (36.2%) 2- Urological: 31 (14.6%) 3- Gynecologic: 24 (11.3%) 4- Colorectal: 22 (10.3%) 5- Lung: 17 (8%)	1 − 0–1 (Active): 95 (44.6%) 2–2 (Ambulatory, < 50% in bed): 80 (37.6%) 3–3 (Limited, > 50% in bed): 24 (11.3%) 4–4 (Disabled/Bedbound): 14 (6.6%)	NR	NR	NS	1- Patient Recommended to Continue Meds: 48 (22.5%) 2- Contraindications (Coagulation/Infection): 35 (16.4%) 3- Patient Refusal: 33 (15.5%) 4- Disease Progression: 29 (13.6%) 5- Pain Not Suitable for Intervention: 20 (9.4%)	213 (60.9%)
Linklater 2002 [17]	220	UK and Ireland	NA	NA	NA	NA	NA	NA	NS	NA	NA
Mahmoud 2023 [28]	64	USA	NA	NA	NA	NA	NA	NA	Intrathecal Drug Delivery System	NA	NA
Matsumoto 2024 [14]	199 hospitals	Japan	NA	NA	NA	NA	NA	NA	NS	NA	NA
Nakazawa 2025 [29]	430	Japan	NA	NA	NA	NA	NA	NA	NS	NA	NA
Stearns 2020 [11]	1403	USA	58.8 (12)	609 (43.4%)	1- Lung: 89 (15.0%) 2- Breast: 65 (11.0%) 3- Colon/Rectal: 64 (10.8%) 4- Pancreatic: 49 (8.3%) 5- Prostate: 35 (5.9%) 6- Other/Unknown: 267 (45.1%)	1- ASA III or IV: 591 (91.1%) 2- ASA II: 58 (8.9%)	NR	NR	Intrathecal Drug Delivery System	1- Undertreatment/Lack of Guideline Adherence 2- Perceived Risks of the Procedure 3- Delayed Referral 4- Systemic Opioid Reliance	NR
Uehara 2022 [18]	1642	Japan	NA	NA	NA	NA	NA	NA	NS	NA	NA
Warner 2019 [13]	120	USA	NA	NA	NA	NA	NA	NA	Intrathecal Drug Delivery System	NA	NA
Brown 1997 [26]	292	USA	NR	157 (53.8%)	Pancreatic Cancer: 292 (100%)	NR	NR	NR	Neurolytic Celiac Plexus Block (NCPB).	NR	NR

Study ID	Provider & Facility Survey Variables				
	Specialty	Experience Level	Training Status	Facility Type	Perceived Barriers
Barnosky 2025 [2]	1- Oncology (*n* = 8) 2- Palliative Care (*n* = 2) 3- Neurosurgery/Pain (*n* = 2) 4- Nurses (*n* = 4) 5- Service Managers (*n* = 2)	13.3 (7.6) in years	NR	1- University-based organization (*n* = 12) 2- District/General Hospital (*n* = 6)	1- Infection risk: 18 (100%) 2- Patient prognosis: 8 (44.4%) 3- Tumor location: 10 (55.6%)
Cahana 2007 [15, 15]	Group 1 “First-line” (4 Doctors, 9 Nurses) Group 2 “Second-line” (4 Support staff) (Chief/Administrative Nurses, Non-certified Nurses (aides), Social Workers, Psychologists, and Physiotherapists)	NS	1- Group 1: 29 (90%) 2- Group 2: 8 (25%)	University Hospital	Technical manipulation difficulty: 14 (43%)
Copley 2025 [27]	Pain Medicine and/or Anesthesia	Consultant clinical lead or most senior clinician in pain medicine and/or Anesthesia	1- Stage 1 & 2: 115 (70%) 2- Stage 3 & SIA (Advanced) 80 (49%)	Mixed Practice Settings	1- Lack of Operating Room (“Theatre”) Space 2- Funding & Equipment Shortages 3- Staffing Vacancies
Goel 2023 [3]	NA	NA	NA	NA	NA
Khashan 2024 [5]	NA	NA	NA	NA	NA
Kim 2020 [20]	NA	NA	NA	NA	NA
Linklater 2002 [17]	Palliative Medicine	Consultants or Medical Directors	15 (7%)	Mixed Practice Settings	1- No formal arrangement: 66 (30%) 2- Geographical isolation: 44 (20%) 5- Lack of Interest: 44 (~ 20%) 4- Lack of Experience: 44 (20%)
Mahmoud 2023 [28]	Pain Medicine and/or Anesthesia	Fellowship Level	48 (64%)	Academic Training Programs	1- Lack of faculty with expertise in IDDS therapy: 21 (33%) 2- Lack of curriculum regarding IDDS: 18 (28%) 3- Not enough IDDS therapy cases at my institution: 18 (28%)
Matsumoto 2024 [14]	Pain Medicine and/or Anesthesia	NS	1- Epi: 111 (55.8%) 2- CPN: 82 (41.2%) 3- Phenol Saddle: 75 (37.7%) 4- IA: 61 (30.7%)	University Hospitals and Postgrad Training Hospitals	1- Technical Skill: No/few physicians technically able: 159 (35.8%) 2- Workforce/Policy: Capable but not permitted: 144 (32.4%) 3- Equipment: Lack of facilities/chemicals: 96 (21.6%) 4- Logistics/Follow-up: Inability to follow up: 95 (21.4%) 5- Complications: Inability to respond to complications: 80 (18.0%) 6- Regional Info: Lack of referral facility info: 70 (15.8%) 7- Cooperation: Lack of department cooperation: 66 (14.9%) 8- Patient Flow: No referrals of eligible patients: 63 (14.2%) 9- Indications: Unable to determine indications: 62 (14.0%) 10- Regional Facilities: No regional referral facilities: 55 (12.4%) 11- Permission: Not permitted at facility: 44 (9.9%) 12- Profitability: Unprofitability of procedure: 44 (9.9%)
Nakazawa 2025 [29]	1- Internal Medicine: 253 (58.8%) 2- Surgery: 90 (20.9%) 4- Other: 82 (19.1%) 3- Anesthesia/Palliative Care: 5 (1.2%)	NS	Basic Palliative Care Training Completed: 299 (69.5%)	NS	1- Procedural Gaps: Hospitals with fewer cancer deaths (< 50) lack the expertise to determine eligibility for nerve blocks or radiation therapy, leading to higher consultation needs. 2- Medication Hurdles: Clinicians frequently require guidance on medication selection and unfamiliar prescriptions.
Stearns 2020 [11]	NA	NA	NA	NA	NA
Uehara 2022 [18]	1- Pain Medicine and/or Anaesthesia 2- Home Hospice care 3- Oncology	NS	NS	Designated Cancer Hospitals/University Hospitals	1- Designated Cancer Hospitals/University Hospitals 2- Lack of Time 3- Institutional Permission 4- Communication
Warner 2019 [13]	1- Hospice Nurses: 37 (35.9%) 2- Medical Directors: 18 (17.5%) 3- Nursing Coordinators: 12 (11.7%) 4- Hospice Staff Physicians: 8 (7.8%) 5- Nurse Practitioners: 7 (6.8%) 6- Other: 21 (20.4%)	Hospice Experience: 11.2 (6.8)	NS	1- Home Hospice: 65 (77.4%) 2- Inpatient/Residential Hospice: 10 (11.9%) 3- Other: 9 (10.7%)	1. Provider Confidence and Knowledge Deficits 2- Vendor Unavailability: More than half of the respondents (55%) were unable to identify a vendor to assist with home-based pump maintenance 3- Preference for Systemic Therapy: The vast majority of providers—83.3% (75/90)—preferred using systemic pharmacotherapy (oral/IV opioids)
Brown 1997 [26]	1- Gastroenterologists: 31 (52.5%) 2- Medical Oncologists: 15 (25.4%) 3- General Surgeons: 13 (22%)	NR	NR	Tertiary Care	1- Limited appointment availability: 14 (40%) 2- Need for repeating procedure: 5 (14%) 3- Lack of long-term efficacy: 3 (9%) 4- Prefer alternative therapy: 3 (9%) 5- High cost: 1 (3%) 6- Patient lack of acceptance: 1 (3%) 7- Other: 8 (23%)

### Risk of bias

Observational studies were assessed using the appropriate NIH Quality Assessment Tools (Cohort and Cross-Sectional versions), which evaluated design-specific elements such as population representativeness, exposure and outcome measurement, control for confounding variables, and statistical rigor. These studies received ratings of “Good,” “Fair,” or “Poor,” corresponding to low, moderate, or high risk of bias, respectively. Twelve studies were rated as fair, one was of good quality, and one was rated as poor quality Table S1.

### Results of syntheses

#### Barriers to pain management with Intrathecal Drug Delivery Systems (IDDS)

**Fig. 2:**
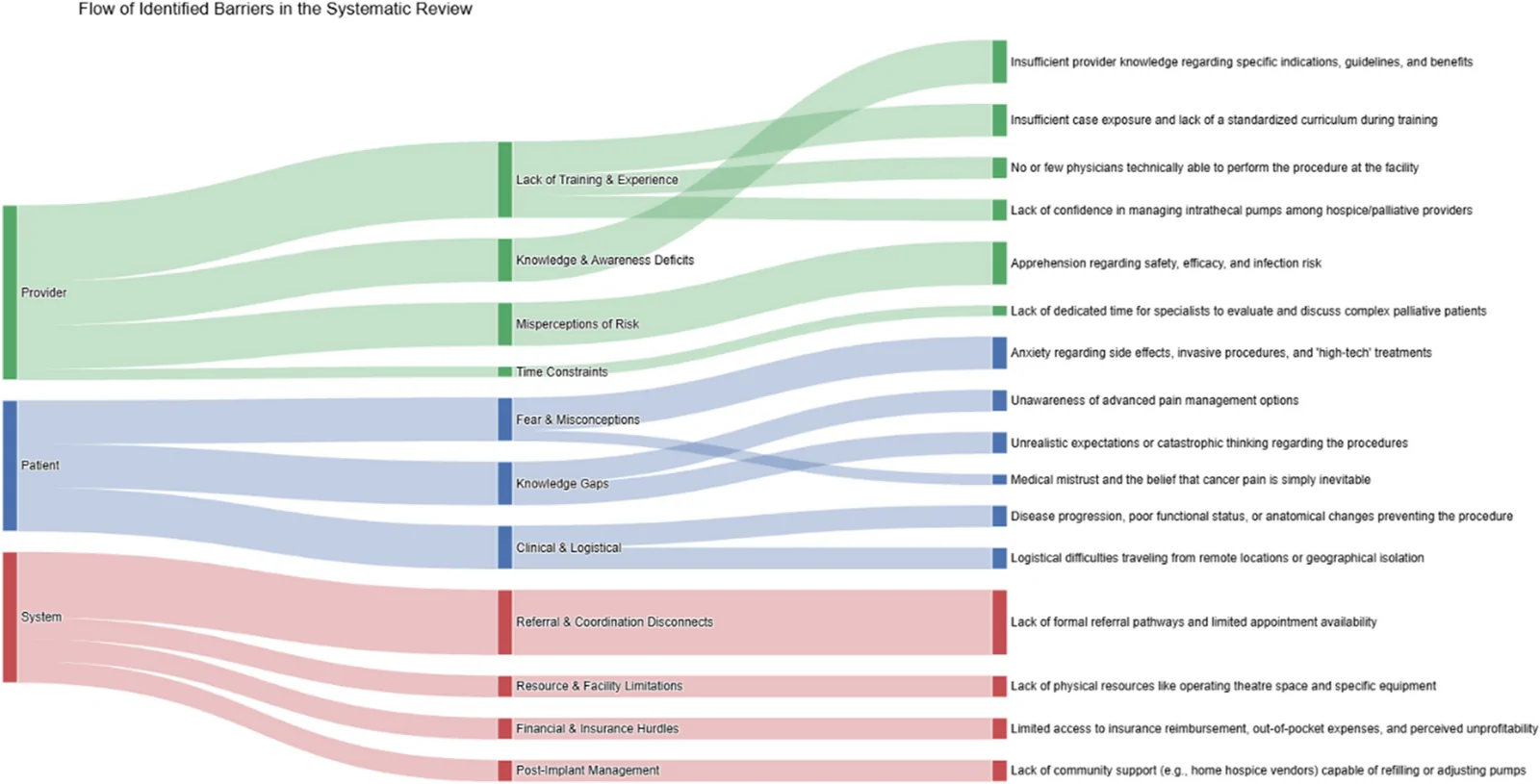
Sankey Diagram Mapping Categories of Barriers to Pain Management Interventions

Eight studies addressed the barriers that limit access to IDDS as a pain management option for cancer patients **(**Fig. 2; Table 2**)** [2, 3, 11, 13–15, 18, 28].

**Table 2:** Summary of the Barriers to Pain Management Interventions

Level	Category	Specific Barrier	Sources
Patient	Fear & Misconceptions	Anxiety regarding side effects, invasive procedures, and ‘high-tech’ treatments	Barnosky 2025 [2], Cahana 2007 [15], Kim 2020 [20]
		Medical mistrust and the belief that cancer pain is simply inevitable	Goel 2023 [3]
	Knowledge Gaps	Unawareness of advanced pain management options	Barnosky 2025 [2], Kim 2020 [20]
		Unrealistic expectations or catastrophic thinking regarding the procedures	Cahana 2007 [15], Khashan 2024 [5]
	Clinical & Logistical	Disease progression, poor functional status, or anatomical changes preventing the procedure	Khashan 2024 [5], Kim 2020 [20]
		Logistical difficulties traveling from remote locations or geographical isolation	Khashan 2024 [5], Linklater 2002 [17]
Provider	Lack of Training & Experience	Insufficient case exposure and a lack of a standardized curriculum during training	Copley 2025 [27], Mahmoud 2023 [28], Matsumoto 2024 [14]
		No or few physicians are technically able to perform the procedure at the facility	Matsumoto 2024 [14], Nakazawa 2025 [29]
		Lack of confidence in managing intrathecal pumps among hospice/palliative providers	Goel 2023 [3], Warner 2019 [13]
	Knowledge & Awareness Deficits	Insufficient provider knowledge regarding specific indications, guidelines, and benefits	Barnosky 2025 [2], Khashan 2024 [5], Linklater 2002 [17], Matsumoto 2024 [14]
	Misperceptions of Risk	Apprehension regarding safety, efficacy, and infection risk	Barnosky 2025 [2], Brown 1997 [26], Cahana 2007 [15], Goel 2023 [3]
	Time Constraints	Lack of dedicated time for specialists to evaluate and discuss complex palliative patients	Matsumoto 2024 [14]
System	Resource & Facility Limitations	Lack of physical resources, such as operating theatre space and specific equipment	Copley 2025 [27], Matsumoto 2024 [14]
	Referral & Coordination Disconnects	Lack of formal referral pathways and limited appointment availability	Barnosky 2025 [2], Brown 1997 [26], Copley 2025 [27], Khashan 2024 [5], Linklater 2002 [27], Nakazawa 2025 [29]
	Financial & Insurance Hurdles	Limited access to insurance reimbursement, out-of-pocket expenses, and perceived unprofitability	Goel 2023 [3], Matsumoto 2024 [14]
	Post-Implant Management	Lack of community support (e.g., home hospice vendors) capable of refilling or adjusting pumps	Matsumoto 2024 [14], Warner 2019 [13]

### Patient-related barriers

Patients often expressed a lack of understanding regarding how intrathecal pumps manage refractory pain [2, 3]. In addition, there was a fear of infection among immunocompromised patients, and other perceived risks associated with implantation [2, 3, 11]. There were attitudinal and psychological barriers as well, including misconceptions about opioids, medical mistrust, and a fear of distracting physicians from primary cancer treatment [3, 15]. In one study, patients exhibited negative attitudes towards high-tech interventions, which were perceived to be giving false hope or unrealistic expectations at the end of life [15].

### Clinician-related barriers

A significant lack of standardized curriculum and faculty expertise. Specifically, 51% of pain medicine fellows reported an unmet need for training, with one-third participating in five or fewer implants during residency [13, 28]. In another study, respondents indicated that there were no or few physicians able to perform this procedure in their facility. Others cited the inability to follow up or to determine whether the procedure is indicated [14]. Variability in how clinicians evaluate refractory pain and a lack of consensus on the estimated life expectancy required to qualify for an implant [2].

In one study, only 28% of hospice providers felt confident managing patients with existing pumps, leading to a strong preference (> 80%) for systemic analgesics over interventional therapies [13]. Such attitudinal resistance was evident in the way caregivers often perceived technical device manipulation as difficult (43% in one study) [15], or view the involvement of too many specialists as futile [15].

### Systemic and administrative barriers

Interdisciplinary coordination failures caused a disconnect between oncology, pain management, and neurosurgery regarding responsibility for the patient [2]. Uncooperative work environment between departments was also a barrier [14, 18]. This was compounded by a lack of structured clinical guidelines and a failure to include interventional pain plans within Survivorship Care Plans [2, 11, 13]. Moreover, there was a lack of referrals, or a median delay of over two years from cancer diagnosis to IDDS implant, resulting in prolonged periods of uncontrolled pain [11, 14]. This was made worse by systemic disruptors, such as the COVID-19 pandemic [2]. Some of the reported logistical limitations included difficulties in dose titration, refilling, and accessing vendors for maintenance, especially in home care or hospice settings [2, 13].

### Socioeconomic and demographic barriers

Data indicate that access to IDDS is not equitable across different populations. For instance, patients from racial minorities are significantly less likely to receive IDDS implants compared to Caucasian patients [3]. In addition, residents of lower-income neighborhoods reportedly faced higher barriers to access than those in affluent areas [3]. This was likely due to the high upfront cost of the device and procurement compared to traditional treatments, which remains a significant technical and administrative limitation [2, 14].

#### Barriers to pain management with Celiac Plexus Neurolysis (CPN)

Three studies provided data regarding barriers to pain management with CPN **(**Fig. 2; Table 2**)** [14, 18, 26]. Patient-related barriers were primarily due to patient refusal to receive CPN [26].

### Clinician-related barriers

Among clinicians, there were misconceptions regarding the safety of the procedure, which prevented its implementation [26]. Moreover, there was a lack of the necessary skills to determine whether the procedure is indicated, to perform the procedure, follow up after the procedure, or respond to procedural complications when they arise [14]. This was worsened by the limited number of cases, which caused difficulties in skill acquisition [18].

### Systemic and administrative barriers

In one study, technically capable physicians were not allowed to perform the procedure due to work-related restrictions [14]. Moreover, uncooperative relevant medical departments prevented the implementation of the procedure [14, 18]. Other systemic issues included the unavailability of equipment, facilities, or chemicals required for the procedure [14]. Two studies reported that the procedure was not allowed to be performed at their facility [14, 18], possibly due to unprofitability [14].

### Barriers to pain management with various methods

Six studies addressed barriers to various methods of pain management in general, such as phenol saddle block, epidural infusions, and other interventional pain procedures (IPPs; Fig. 2; Table 2) [5, 14, 18, 20, 27, 29].

### Patient-related barriers

Psychological barriers included significant fear and anxiety regarding invasive procedures, side effects, and general resistance to invasive therapy. On the other hand, there were high rates of lost opportunities (24% in one study), where patients deteriorated or passed away before the scheduled intervention [5]. The functional status of patients also limited the use of some interventions due to inability to assume necessary positions for the procedure, coagulopathy, or active infections. In addition, a lack of information provided to the patient regarding the procedure’s nature and benefits discouraged patients from undergoing the procedures.

### Clinician-related barriers

Referring physicians often lacked awareness regarding patient eligibility and expected outcomes for ablative procedures. Furthermore, there was a widespread absence of basic palliative care training and limited clinical experience, particularly in non-specialist hospitals. A high percentage of facilities report having few or no physicians technically capable of performing specific procedures like Phenol Saddle Blocks. There was also a lack of interest among some palliative consultants and a limited perception of the roles that interventional specialists (like anesthetists) should play in prescribing these procedures.

#### Systemic and administrative barriers

Effective patient care for refractory pain is currently hindered by an interplay of systemic and structural barriers. At the foundational level, coordination failures, characterized by a lack of formal arrangements between palliative and pain specialists, are exacerbated by a lack of international consensus on defining refractory pain, which makes standardized referrals nearly impossible. These administrative hurdles are compounded by logistical and geographical challenges, where patient isolation and the distance from specialized centers create significant access gaps. Even when patients reach a facility, they face staffing and infrastructure shortages, including a lack of specialized psychologists, limited theater space, and scheduling conflicts with concurrent treatments like chemotherapy. Furthermore, these issues are intensified by financial disincentives for certain procedures and commissioning deficits in cancer services, all of which were worsened by external pressures like the COVID-19 pandemic. These constraints result in an inability to provide essential post-procedure follow-up, leaving patients vulnerable if complications arise.

## Discussion

Our systematic review revealed that barriers to interventional pain management, particularly for intrathecal drug delivery systems (IDDS) and celiac plexus neurolysis (CPN), extend well beyond the patient level. While patient-related factors such as fear, knowledge gaps, and medical deterioration are significant, the synthesized data indicate that clinician and systemic factors are also substantial obstacles. Clinician-related barriers are pronounced for IDDS, where a striking 51% of pain management fellows report unmet training needs, and most hospice providers lack confidence in managing these devices. This deficiency in education and hands-on experience creates a cycle of low referral rates and clinical uncertainty, delaying patient access to effective therapy. The data on CPN highlighted an inability to determine the indication or perform the procedure as core clinician hurdles.

Sun et al. reported several patient barriers to pain management. For instance, patients were concerned that pain could not be managed. Patients also believed that pain medication was addictive, especially in cancer patients. Moreover, patients feared that relieving pain would make it difficult for doctors to detect health deterioration. Some of the barriers included fear of inconveniencing doctors with complaints about pain, and if they focus on dealing with the pain, they would not be paying attention to curing their disease. Other barriers included misconceptions about side effects, such as constipation and psychiatric adverse effects [21].

Systemic and administrative failures further compound these issues. The lack of structured clinical guidelines, coupled with poor coordination between oncology, pain, and surgical services, creates a fragmented care pathway. The reported median delay of over two years from diagnosis to IDDS implant and the high rate (24%) of patient deterioration before intervention are direct consequences of these structural inefficiencies. Logistical challenges in device maintenance and follow-up, particularly in homecare settings, and external disruptive factors like the COVID-19 pandemic, exacerbate this fragility. Furthermore, socioeconomic and racial disparities confirm that even when clinical barriers are overcome, systemic biases prevent a significant portion of the patient population from benefiting from these technologies.

The disruption to health systems and subsequent staffing challenges caused by the COVID-19 pandemic overshadowed and possibly deprioritized the adoption of new pain control methods, including IDDS. These barriers resulted in avoidance behaviors among both current users and non-users of these devices [2]. In addition, the pandemic limited the exposure of clinicians to patients, lowering the number of training opportunities for pain medicine fellows learning to perform interventional pain procedures [28].

In a qualitative study conducted in Iran, Orujlu et al. explored why cancer pain management often falls short, identifying barriers that span individual beliefs, healthcare provider attitudes, and systemic failures. The researchers identified barriers through interviews with 14 patients. For many patients in this study, religious beliefs played a central role in how they perceived and managed, or failed to manage, their pain. Some patients believed that their pain was a punishment, a blessing, or an atonement for sins. These beliefs led patients to be apathetic towards seeking pain management. Other patients expressed negative attitudes towards pain medication for fear of addiction and side effects, or felt the drugs were simply ineffective, leading them to prefer traditional herbal decoctions over pharmacological interventions [19].

Our synthesis identified hesitation to perform pain procedures as a clinician-level barrier. Woodrow et al. demonstrated that graduating pain medicine fellows report low post-graduation comfort for several cancer-specific procedures, including intrathecal pump implantations (31.3%) and vertebral body radiofrequency ablations (16.7%). This diminished comfort correlates directly with low procedural volume. This lack of comprehensive exposure correlates directly with low procedural volume and functions as a systemic barrier, limiting procedural versatility in independent practice [30].

To break these barriers, overcoming technical apprehension among staff requires hands-on coaching. One study found that after simple hands-on instruction, caregivers viewed initially difficult techniques as feasible [15]. For hospice providers, desired educational formats include lunch and learn conferences, electronic curricula, and formal hands-on training [13]. Khashan et al. organized an interdisciplinary team consisting of a palliative care specialist, a nurse, and a neurosurgeon to assess cancer patients for eligibility for interventional pain management. This team provided patients with more comprehensive insights regarding their pain management options. In addition, this allows for a collaborative approach to managing pain in patients with complex disease features, optimizing overall care, as each specialist uses their expertise in selecting the best method of palliation, whether minimally invasive procedures through pain specialists or neuro-ablative procedures done by the neurosurgeon [5]. Corriero et al. indicated that interventional pain management procedures offer effective analgesia with fewer systemic effects, but are underutilized due to delayed referrals and limited awareness. They reported that emerging technologies like AI-driven decision support and automated referrals present opportunities to overcome these barriers, enabling earlier, individualized intervention, and integrating interdisciplinary collaboration and technological innovations to ensure timely and effective pain relief [9].

This is the first systematic review to address barriers to interventional pain management procedures, such as IDDS and CPN. However, we could not perform a proper analysis due to the qualitative nature of most studies. Future studies should rigorously assess the barriers that limit access to pain management interventions for cancer patients at different stages of their disease, introduce targeted approaches to overcome these barriers to improve the outcomes for cancer patients, especially those who are receiving end-of-life care.

## Conclusion

In conclusion, this systematic review demonstrated that the underutilization of interventional pain management techniques like IDDS and CPN is not driven by a single factor but by a complex interplay of patient misconceptions, clinician training gaps, and systemic fragmentation. While patient fears and beliefs remain significant, the primary obstacles are the lack of standardized education for providers and the absence of integrated, multidisciplinary care pathways. These inefficiencies lead to unacceptable delays and inequitable access. Overcoming this challenge requires a shift from isolated practice to a collaborative model, incorporating structured interdisciplinary teams, mandatory hands-on training, and the integration of technological innovations to ensure timely, equitable, and effective pain relief for all patients.

## Supplementary Information

Supplementary Material 1. Table S1: Risk of Bias for Cross-Sectional Studies.

## Data Availability

All data generated or analyzed during this study are included in this published article and its supplementary information files. The datasets used during this study are available from the corresponding author upon request.
