# Supplementary material for: Barriers to interventional pain management in patients with cancer: a systematic review
Source: BMC Palliat Care. 2026 Jul 14;25:296. doi: 10.1186/s12904-026-02235-5 (PMC13647747; doi:10.1186/s12904-026-02235-5)
Supplement: Supplementary file 1 [file 12904_2026_2235_MOESM1_ESM.docx]

| **Study ID** | **NIH Quality Assessment Tool for Observational Cohort and Cross-Sectional Studies** | | | | | | | | | | | | | | | |  |
| --- | --- | --- | --- | --- | --- | --- | --- | --- | --- | --- | --- | --- | --- | --- | --- | --- | --- |
|  | **1. Was the research question or objective in this paper clearly stated?** | **2. Was the study population clearly specified and defined?** | **3. Was the participation rate of eligible persons at least 50%?** | **4. Were all the subjects selected or recruited from the same or similar populations (including the same time period)? Were inclusion and exclusion criteria for being in the study prespecified and applied uniformly to all participants?** | **5. Was a sample size justification, power description, or variance and effect estimates provided?** | **6. For the analyses in this paper, were the exposure(s) of interest measured prior to the outcome(s) being measured?** | **7. Was the time frame sufficient so that one could reasonably expect to see an association between exposure and outcome if it existed?** | **8. For exposures that can vary in amount or level, did the study examine different levels of the exposure as related to the outcome (eg, categories of exposure, or exposure measured as continuous variable)?** | **9. Were the exposure measures (independent variables) clearly defined, valid, reliable, and implemented consistently across all study participants?** | **10. Was the exposure(s) assessed more than once over time?** | **11. Were the outcome measures (dependent variables) clearly defined, valid, reliable, and implemented consistently across all study participants?** | **12. Were the outcome assessors blinded to the exposure status of participants?** | **13. Was loss to follow-up after baseline 20% or less?** | **14. Were key potential confounding variables measured and adjusted statistically for their impact on the relationship between exposure(s) and outcome(s)?** | **total scores** | **Quality rating: good (11-14 points) or fair (7.5-10.5 points) or poor (0-7 points)** | |
|  | **Yes / No / Not reported (NR) or cannot determine (CD) or not applicable (NA)** | **Yes / No / Not reported (NR) or cannot determine (CD) or not applicable (NA)** | **Yes / No / Not reported (NR) or cannot determine (CD) or not applicable (NA)** | **Yes / No / Not reported (NR) or cannot determine (CD) or not applicable (NA)** | **Yes / No / Not reported (NR) or cannot determine (CD) or not applicable (NA)** | **Yes / No / Not reported (NR) or cannot determine (CD) or not applicable (NA)** | **Yes / No / Not reported (NR) or cannot determine (CD) or not applicable (NA)** | **Yes / No / Not reported (NR) or cannot determine (CD) or not applicable (NA)** | **Yes / No / Not reported (NR) or cannot determine (CD) or not applicable (NA)** | **Yes / No / Not reported (NR) or cannot determine (CD) or not applicable (NA)** | **Yes / No / Not reported (NR) or cannot determine (CD) or not applicable (NA)** | **Yes / No / Not reported (NR) or cannot determine (CD) or not applicable (NA)** | **Yes / No / Not reported (NR) or cannot determine (CD) or not applicable (NA)** | **Yes / No / Not reported (NR) or cannot determine (CD) or not applicable (NA)** |  |  |  |
| **Barnosky 2025** | Yes | Yes | NR | Yes | No | NA | NA | NA | Yes | No | Yes | Yes | NA | NA | 7 | Poor | |
| **Brown 1997** | Yes | Yes | Yes | Yes | Yes | Yes | Yes | Yes | Yes | Yes | Yes | No | NA | No | 12 | Good | |
| **Cahana 2007** | Yes | Yes | No | Yes | No | No | Yes | Yes | Yes | No | No | No | No | No | 10 | Fair | |
| **Copley 2025** | Yes | Yes | No | Yes | No | No | NA | Yes | Yes | No | Yes | No | NA | No | 9 | Fair | |
| **Goel 2023** | Yes | Yes | Yes | Yes | No | No | NA | No | Yes | No | Yes | No | NA | No | 9 | Fair | |
| **Khashan 2024** | Yes | Yes | Yes | Yes | No | No | NA | No | Yes | No | Yes | No | Yes | No | 10 | Fair | |
| **Kim 2020** | Yes | Yes | Yes | Yes | No | No | NA | No | Yes | No | Yes | No | NA | No | 9 | Fair | |
| **Linklater 2002** | Yes | Yes | Yes | Yes | No | No | NA | No | No | No | No | No | No | No | 8.5 | Fair | |
| **Mahmoud 2023** | Yes | Yes | Yes | Yes | No | No | NA | No | Yes | No | Yes | NA | No | No | 9 | Fair | |
| **Matsumoto 2024** | Yes | Yes | No | Yes | No | No | NA | No | Yes | No | Yes | No | NA | No | 8.5 | Fair | |
| **Nakazawa 2025** | Yes | Yes | No | Yes | Yes | No | NA | Yes | Yes | No | Yes | No | NA | Yes | 10 | Fair | |
| **Stearns 2020** | Yes | Yes | NR | Yes | No | Yes | Yes | No | Yes | No | Yes | No | Yes | No | 10.5 | Fair | |
| **Uehara 2022** | Yes | Yes | No | Yes | No | No | NA | Yes | Yes | No | Yes | No | NA | Yes | 9.5 | Fair | |
| **Warner 2019** | Yes | Yes | Yes | Yes | No | No | NA | No | Yes | No | Yes | No | NA | No | 9 | Fair | |
